# Single-Cell Transcriptomics Reveals Stem Cell-Derived Exosomes Attenuate Inflammatory Gene Expression in Pulmonary Oxygen Toxicity

**DOI:** 10.3390/ijms26094462

**Published:** 2025-05-07

**Authors:** Jing Shi, Yabin Li, Houyu Zhao, Chenyang Yan, Ruxia Cui, Yukun Wen, Xuhua Yu, Wei Ding, Yunpeng Zhao, Yiqun Fang

**Affiliations:** 1 Naval Medical Center, Naval Medical University, Shanghai 200433, China; fdshijing_sj@126.com (J.S.); zhaohouyuecho@163.com (H.Z.); m18001867308@163.com (Y.W.); xuhua_0813@163.com (X.Y.); 13255589299@163.com (W.D.); 2 Translational Medical Research Center, Naval Medical University, Shanghai 200433, China; wjczhuafanwang@163.com; 3 Department of Life Science and Technology, Tongji University, Shanghai 200092, China; 18127@tongji.edu.cn; 4 College of Biology and Environmental Science, Jishou University, Jishou 416000, China; m15729590521@163.com

**Keywords:** single-cell transcriptomics, cell-derived exosomes, pulmonary oxygen, inflammatory, mesenchymal stem cell exosomes

## Abstract

In recent years, the role played by exosomes in lung diseases has been investigated. Exosomes have been shown to contribute to reductions in lung inflammation and pulmonary fibrosis. However, the role played by exosomes in pulmonary oxygen toxicity and the mechanism involved have not yet been reported. In the present work, we aimed to investigate the mechanism by which stem cell exosomes protect lung tissue and the potential molecular regulatory network involved. In this study, we employed single-cell RNA sequencing techniques to elucidate the unique cellular and molecular mechanisms underlying the progression of exosome therapy for pulmonary oxygen toxicity. We found changes in cell populations after exosome treatment, characterized by the expression of different molecular markers. We also integrated single-cell RNA sequencing (scRNA-seq) and bulk analysis to identify the protective effects of mesenchymal stem cell exosomes (MSC-Exos) in a mouse pulmonary oxygen toxicity (POT) model. scRNA-seq revealed dynamic shifts in the lung cellular composition after exosome treatment, including a reduction in inflammatory lymphoid cells (NK, B cells, CD8^+^ T, CD4^+^ T) and restored alveolar epithelial populations (AT1/AT2). A comprehensive gene expression analysis showed that inflammatory pathways associated with oxidative stress were significantly upregulated. In addition, our analysis of the intercellular interaction network revealed that there was a significant reduction in intercellular signal transduction in the POT group compared to the exosome-treated group. These results not only shed light on the unique cellular heterogeneity and potential pathogenesis following exosome therapy, but they also deepen our understanding of molecular pathophysiology and provide new avenues for targeted therapeutic strategies.

## 1. Introduction

Professional or commercial diving is a global industry that employs tens of thousands of people around the world in a wide variety of jobs [1]. As renewable energy sources increase and the world enters the age of digital connectivity, more and more resources are being deployed under the sea [2]. Regardless of whether a dive is recreational or professional, breathing compressed air underwater exposes divers to several health hazards that are unique to diving [3]. Two of the most notorious diving-related conditions are decompression sickness and barotrauma, which is trauma caused by rapid changes in pressure. Barotrauma can lead to pneumothorax, subcutaneous and mediastinal emphysema, and even to arterial gas embolism that blocks coronary, cerebral, or other end-organ arteries [4]. Less well known is the fact that adverse reactions to breathing gases can also cause serious health concerns for divers [5]. One such concern is oxygen poisoning.

Pathological damage caused by high concentrations of oxygen exposure is primarily characterized by the destruction of the alveolar epithelial barrier, persistent inflammatory responses, and the progression of fibrosis. This condition is commonly observed in scenarios of high oxygen exposure, such as hyperbaric oxygen therapy, mechanical ventilation, and diving medicine. Based on the clinical progression and differences in oxygenation indices (PaO_2_/FiO_2_, where PaO_2_ refers to the arterial oxygen partial pressure and FiO_2_ refers to the fractional inspired oxygen), such damage can be classified into acute lung injury, where the PaO_2_/FiO_2_ ratio ranges from 200 to 300 mmHg (26.7 to 40 kPa), presenting as acute pulmonary edema and diffusion dysfunction, and pulmonary oxygen toxicity, where the PaO_2_/FiO_2_ ratio remains below 200 mmHg (26.7 kPa) [6,7], accompanied by irreversible pulmonary interstitial fibrosis. Clinically, patients may experience symptoms such as retrosternal burning sensation, cough, chest pain, and shortness of breath [8]. In current clinical practice, the management of POT faces two main challenges [9]: on the one hand, the pathological mechanisms are complex, involving oxidative stress, mitochondrial dysfunction, and abnormal activation of the NF-κB signaling pathway; on the other hand, traditional anti-inflammatory and antioxidant therapies have limited efficacy in reversing the fibrotic process [10]. In addressing POT induced by high concentrations of oxygen exposure, there is an urgent need to explore novel therapeutic strategies that effectively target its complex pathological mechanisms and improve patient outcomes.

Mesenchymal stem cells (MSCs) are widely regarded as a promising therapeutic strategy for tissue repair due to their exceptional regenerative abilities and immunomodulatory characteristics [11]. Notably, the reparative functions of MSCs are not solely dependent on their intrinsic biological actions but are significantly regulated by paracrine signals mediated through the extracellular vesicles they secrete, particularly exosomes. Exosomes are small vesicles with a diameter of 40–100 nanometers, encapsulated by a lipid bilayer, containing biomolecules such as proteins, mRNA, miRNA, and DNA [12,13]. These molecules can be transferred between cells, facilitating genetic information exchange, host cell reprogramming, and intercellular communication [14]. Due to their small size, stable structure, low immunogenicity, and good biocompatibility, exosomes have emerged as a highly promising therapeutic tool, demonstrating significant efficacy in the treatment of respiratory diseases, neurological disorders, and cancer. Research has shown that exosomes derived from MSC-Exos can effectively inhibit tumor growth and metastasis by modulating the tumor microenvironment, thus enhancing the efficacy of radiotherapy and chemotherapy [15]. Additionally, MSC-Exos therapy has been validated to effectively alleviate hyperoxia-induced inflammation and immune responses, improve lung function compromised by bronchopulmonary dysplasia, reduce fibrosis and pulmonary vascular remodeling, and mitigate pulmonary hypertension [16]. Furthermore, MSC-Exos can significantly suppress the expression of the pro-inflammatory cytokines IL-6 and TNF-α in lung tissue by delivering active components such as miRNA-21a-5p while simultaneously promoting the regeneration of alveolar epithelium, thereby effectively improving the symptoms of acute lung injury [17]. Despite these findings indicating the immense potential of MSC-Exos-based targeted therapies in treating pulmonary diseases, the specific mechanisms underlying their action within the context of POT remain inadequately elucidated.

With the rapid development of high-throughput sequencing technology, single-cell RNA sequencing (scRNA-seq) has emerged as a core technique for unraveling cellular heterogeneity and molecular dynamics in pathological processes [18]. Its single-cell resolution can precisely reveal the transcriptomic heterogeneity of subpopulations such as alveolar epithelial cells, immune cells, and stromal cells within the lung injury microenvironment, providing a multidimensional molecular map for understanding disease mechanisms. However, there remains a significant research gap regarding the systematic analysis of the molecular regulatory network of MSC-Exos in the context of POT based on scRNA-seq technology. This study aims to construct a mouse model of POT induced by hyperoxia, followed by tail vein injection of MSC-Exos for pre-treatment, to quantitatively assess the levels of inflammatory factors in lung tissue and verify their anti-inflammatory effects. Furthermore, we will utilize scRNA-seq technology to construct a single-cell transcriptomic profile of lung tissue, analyzing the gene expression reprogramming characteristics of major cell subpopulations under MSC-Exos intervention, and screen for key pathways through kyoto encyclopedia of genes and genomes (KEGG) enrichment analysis. Through these research methods and analytical strategies, we aim to reveal the cell-specific molecular networks in POT and provide important theoretical support for the effective translation of exosome therapies into clinical applications.

## 2. Results

### 2.1. MSC-Exos Can Alleviate the Inflammatory Response Induced by a Model of Pulmonary Oxygen Toxicity

**Figure 1:**
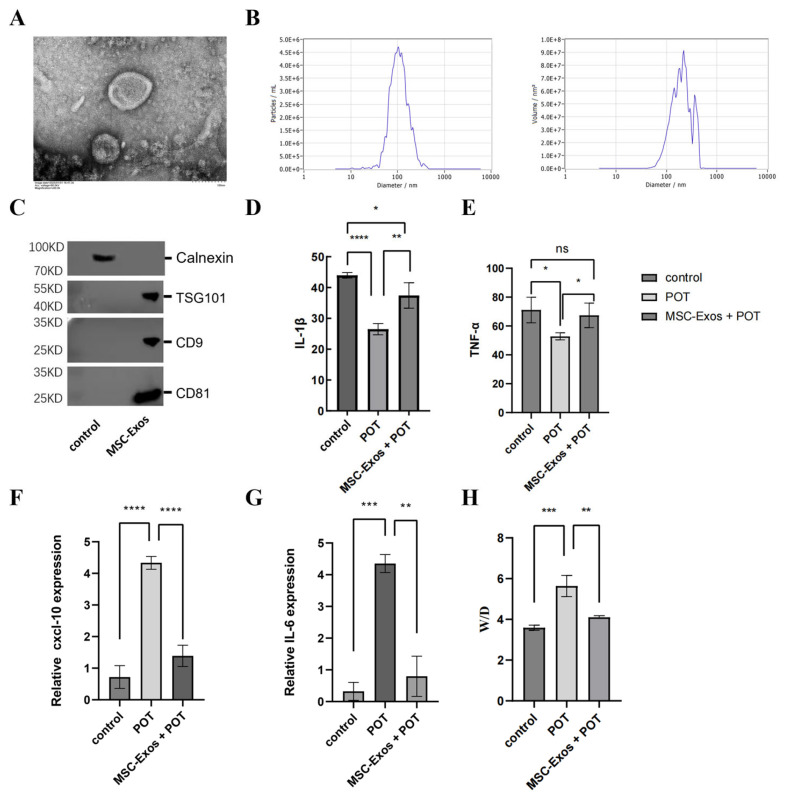
(**A**) Morphology of MSC-Exos determined using TEM (scale bar = 200 nm). (**B**) MSC-Exos size determined using nanoparticle tracking analysis. (**C**) Analysis of exosome-specific proteins CD9, CD81, and TSG101 using western blotting. (**D**) IL-1β levels in mouse lung tissue were quantified by ELISA. (**E**) TNF-α levels in mouse lung tissue were quantified by ELISA. (**F**) mRNA levels of *CXCL10* in mouse lung tissues. (**G**) mRNA levels of *IL-6* in mouse lung tissues. (**H**) The edema index of mouse lung tissue (Wet/Dry, W/D). Data are presented as mean ± standard deviation. The notation “ns” signifies no significant difference, the following indicators represent statistical significance: * *p* < 0.05, ** *p* < 0.01, *** *p* < 0.001, **** *p* < 0.0001. Sections (**D**–**H**) were analyzed using one-way ANOVA followed by Tukey’s multiple comparisons test.

In accordance with the method established in a preceding study, MSC-Exos were isolated and their biological characteristics identified using a cryo-transmission electron microscope (Cryo-TEM). The results of this analysis revealed the presence of cup-like spherical vesicles (Figure 1A). The results of the nanoparticle tracking analysis demonstrated that the MSC-Exos exhibited a mean diameter of 105.2 ± 30.1 nm (mean ± standard deviation), indicating the presence of small microvesicle-like particles (Figure 1B). The MSC-Exos under investigation expressed the proteins TSG101, CD9, and CD81 but were negative for calnexin and cytochrome C (Figure 1C). To clarify the protective effect of exosome treatment against hyperbaric lung injury, we injected MSC-Exos into a mouse model of POT. The ELISA results from mouse lung tissues showed that, compared to the control group, the expression levels of IL-1β and TNF-α were significantly downregulated in the POT group, while treatment with MSC-Exos reversed this change (*p* < 0.05) (Figure 1D,E). Gene expression analysis indicated that, relative to the control group, the mRNA levels of CXCL10 and IL-6 were significantly increased in the lung tissues of the POT group, whereas MSC-Exos intervention significantly inhibited this increase (*p* < 0.05) (Figure 1F,G), Meanwhile, the results of weighing the lung tissues from each group of mice showed that the edema index in the POT group was significantly increased compared to the control group, while the edema index decreased following MSC-Exos intervention (*p* < 0.05) (Figure 1H). These results indicate that the exosomes significantly alleviated pulmonary inflammatory damage.

### 2.2. Interactions Among Cells in POT Fibrotic Niche After MSC-Exos Administration

**Figure 2:**
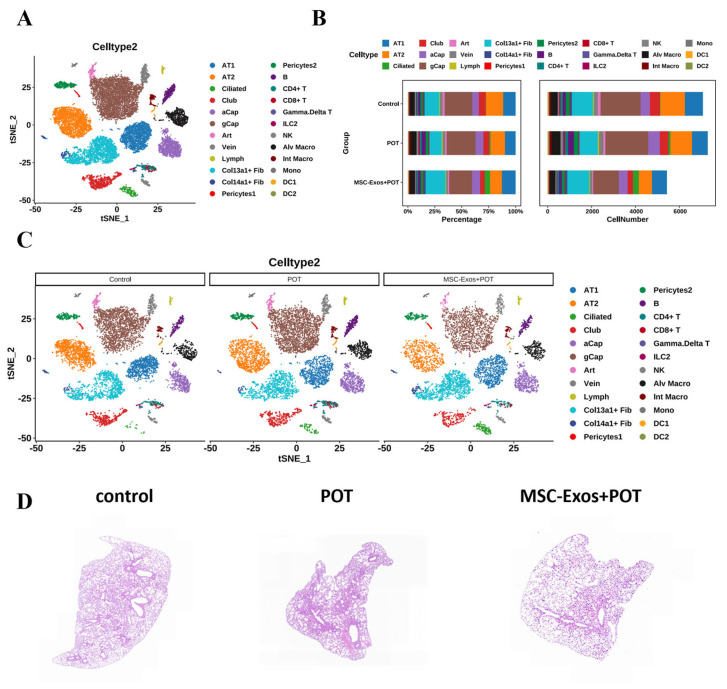
(**A**) Unbiased clustering of 19,792 nuclei from all samples identifies 24 major cell types. (**B**) Relative proportions of each cell type for each condition. + indicates expansion; − indicates contraction (control, POT, MSC-Exos + POT). (**C**) Comparison of nucleus densities in the tSNE space for the three conditions reveals remarkable changes in the relative proportions of cell types. Nuclei were randomly sampled in equal numbers for each group. (**D**) H&E staining images of lung tissues.

In this study, normal and pulmonary-oxygen-poisoning tissues were collected from exosome-treated and untreated mice for single-cell transcriptome sequencing in order to confirm the anti-damage mechanism of exosomes. A total of 19,729 lung cells were divided into three groups after clustering. The dataset of lung histiocytes was annotated using signatures of known lineage markers (Figure 2A), and each marker contained lung cells from cell clusters and lineages in the control, POT, and MSC-Exos + POT groups. Observations were made of dynamic changes to the cellular composition of lungs in the POT model after exosomal treatment, where hyperbaric oxygen-induced lung injury altered the distribution of lung cells. As illustrated in the figure, there was an increase in certain cell types, such as CoI13a1 + Fib, ciliated cells, and AT1 cells, while B cells, NK cells, gCap cells, and Mono cells exhibited a decrease under MSC-Exos + POT conditions (Figure 2B,C). Histological examination with Hematoxylin-eosin (H&E) staining revealed that the POT group mice exhibited pathological features such as inflammatory cell infiltration, interstitial edema, and diffuse alveolar damage in the lung tissue. In contrast, these pathological changes were significantly alleviated following intervention with MSC-Exos (Figure 2D). These results indicate that the effects of hyperbaric oxygen are primarily mediated by inflammatory signals and that MSC-Exos can effectively mitigate the lung damage induced by these signals.

### 2.3. Exosome Treatment Alters AT1 Populations in Mouse Lungs

**Figure 3:**
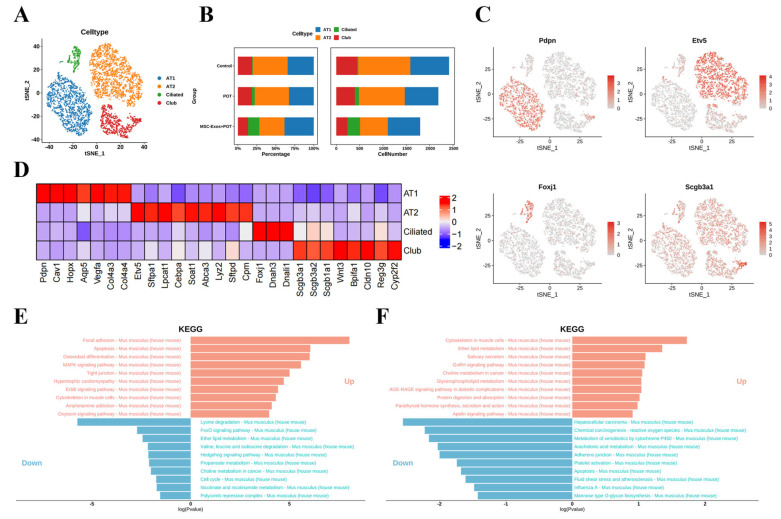
(**A**) Unbiased clustering of 19,792 nuclei from all samples identifies 4 major cell types. (**B**) Comparison of nucleus densities in the tSNE space for the three conditions reveals remarkable changes in the relative proportions of cell types. Nuclei were randomly sampled in equal numbers for each group. (**C**) Biomarker molecules for cell subpopulations. (**D**) Heatmap of the top 10 marker genes in each cluster. (**E**) KEGG enrichment assay pathway (control vs. POT). (**F**) KEGG enrichment assay pathway (POT vs. MSC-Exos + POT).

The lung, as a complex multifunctional organ, is vital to human survival [19]. Lung epithelial stem cells play an important role in lung regenerative repair [20]. In this study, we identified populations of four different epithelial cells, including four bronchial epithelial cells: AT1 cells, AT2 cells, club cells, and ciliated cells (Figure 3A). AT1-like cell populations highly express Pdpn, Cav1, Hopx, AqP5, Vegfa, Col4a3, and Col4a4 (Figure 3B–D).

We conducted a KEGG pathway enrichment analysis of differentially expressed genes. The results showed that in the differential gene enrichment analysis between the POT group and the control group, the apoptosis signaling pathway in alveolar type 1 (AT1) cells was significantly upregulated. In contrast, in the differential gene enrichment analysis between the MSC-Exos + POT group and the POT group, the apoptosis signaling pathway in AT1 cells was significantly downregulated (Figure 3E,F). These results suggest that exosome intervention may protect against hyperbaric oxygen-induced lung injury by suppressing cell apoptosis.

### 2.4. Stem Cell-Derived Exosomes Promote the Expression of Antioxidant Genes in Pulmonary Microvascular Endothelial Cells

**Figure 4:**
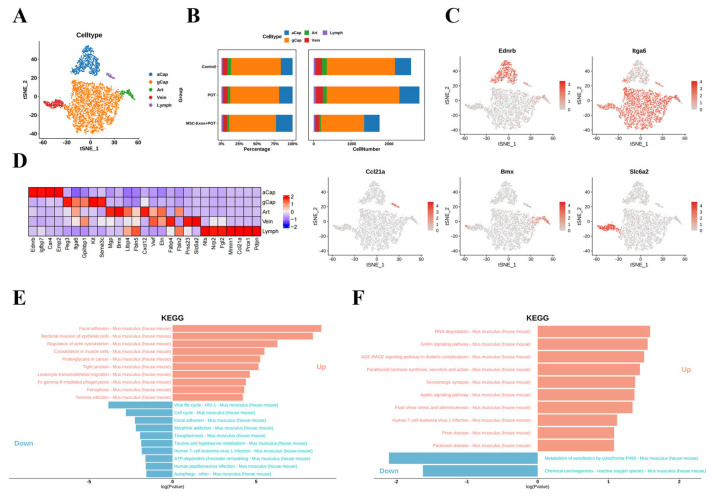
(**A**) Unbiased clustering of 19,792 nuclei from all samples identifies 5 major cell types. (**B**) Comparison of nucleus densities in the tSNE space for the three conditions reveals remarkable changes in the relative proportions of cell types. Nuclei were randomly sampled in equal numbers for each group. (**C**) Biomarker molecules for cell subpopulations. (**D**) Heatmap of the top 10 marker genes in each cluster. (**E**) KEGG enrichment assay pathway (control vs. POT). (**F**) KEGG enrichment assay pathway (POT vs. MSC-Exos + POT).

Based on transcriptional profiles, the authors identified five distinct endothelial cell subtypes, including arterial, venous, capillary, and lymphatic endothelial cells. The capillary endothelial cells were further subdivided into two distinct clusters: general capillaries (gCaps) and alveolar capillaries (aCaps) (Figure 4A). Single-cell analysis revealed that the proportion of gCap cells increased in normal lung tissue, concomitant with a decline in aCap populations. Strikingly, hyperbaric oxygen injury triggered pathological increases in the numbers of both gCap and aCap cells, indicative of disrupted endothelial homeostasis (Figure 4B–D). KEGG enrichment analysis revealed that pulmonary oxygen toxicity induced the upregulation of oxidative pathways in venous vascular endothelial cells. After treatment with stem cell exosomes, some pathways related to reactive oxygen species were downregulated (Figure 4E,F). Based on the above results, we speculate that the regulation of cell apoptosis and the inflammatory response by MSC-Exos may be closely related to the alleviation of intracellular oxidative stress.

### 2.5. Stem Cell-Derived Exosome Therapy Induces Dramatic Shifts in Murine Lung Stromal Col13a1 + Fibroblasts and Pericyte Populations

**Figure 5:**
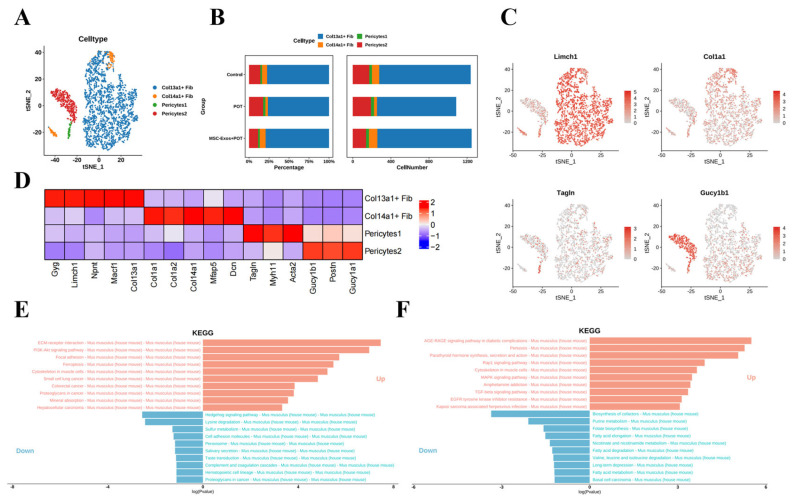
(**A**) Unbiased clustering of 19,792 nuclei from all samples identifies 4 major cell types. (**B**) Comparison of nucleus densities in the tSNE space for the three conditions reveals remarkable changes in the relative proportions of cell types. Nuclei were randomly sampled in equal numbers for each group. (**C**) Biomarker molecules for cell subpopulations. (**D**) Heatmap of the top 10 marker genes in each cluster. (**E**) KEGG enrichment assay pathway (control vs. POT). (**F**) KEGG enrichment assay pathway (POT vs. MSC-Exos + POT).

Within the stroma, we identified four populations, including pericytes and fibroblasts (Figure 5A). We identified two clusters of pericytes, distinguished by the expression of *TagIn* (Pericytes 1) and *Gucy1b1* (Pericytes 2) (Figure 5B–D). The results of the KEGG pathway enrichment analysis indicate that in the comparison between the POT group and the control group, the pathways of Focal adhesion and PlaK-Akt signaling are significantly upregulated. In contrast, these signaling pathways are significantly downregulated in the comparison between the MSC-Exos + POT group and the POT group (Figure 5E,F). These findings suggest that MSC-Exos are associated with the regulation of cell adhesion and the processes of proliferation or apoptosis in pericytes and fibroblasts.

### 2.6. Stem Cell Exosome Treatment Inhibits Lymphoid NK Cells, B Cells, and CD8^+^ and CD4^+^ T Cells in Mice

**Figure 6:**
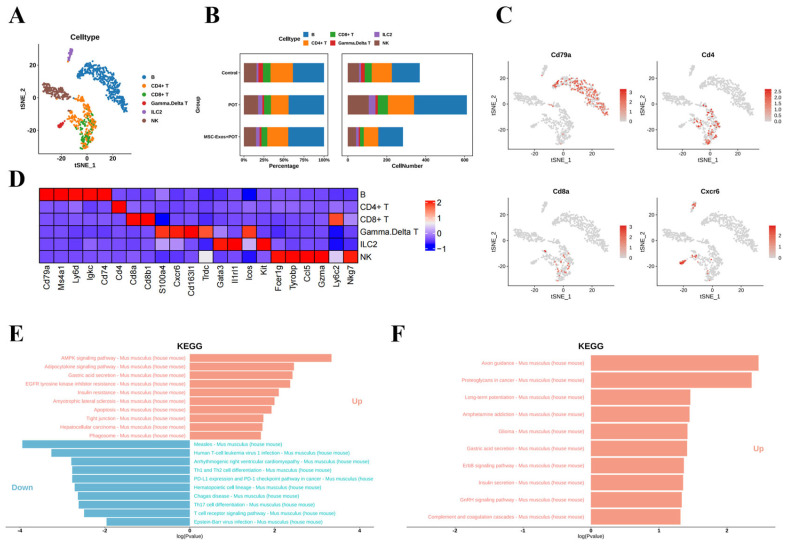
(**A**) Unbiased clustering of 19,792 nuclei from all samples identifies 6 major cell types. (**B**) Comparison of nucleus densities in the tSNE space for the three conditions reveals remarkable changes in the relative proportions of cell types. Nuclei were randomly sampled in equal numbers for each group. (**C**) Biomarker molecules for cell subpopulations. (**D**) Heatmap of the top 10 marker genes in each cluster. (**E**) KEGG enrichment assay pathway (control vs. POT). (**F**) KEGG enrichment assay pathway (POT vs. MSC-Exos + POT).

Based on known cellular markers, we identified six lymphocyte populations, including B cells, T cells, NK cells, and ILC2 cells (Figure 6A–C). The analysis of cell clustering proportions showed that in the MSC-Exos + POT group, the proportions of NK cells, B cells, CD4^+^ T cells, and CD8^+^ T cells significantly decreased compared to the POT group (Figure 6B). The KEGG enrichment analysis of differentially expressed genes revealed that the major pathways significantly affected include those involved in inflammatory responses, the cell cycle, and oxidative phosphorylation (Figure 6E,F). These results indicate that under high oxygen exposure, NK cells, B cells, CD4^+^ T cells, and CD8^+^ T cells in the innate immune system respond first, mediating the inflammatory response.

## 3. Discussion

In recent years, the advent of single-cell RNA sequencing (scRNA-seq) technology has led to a rapid phase of discovery in lung research, including the identification of airway epithelial cells and the characterization of pro-fibrotic macrophages and abnormal basal cells in idiopathic pulmonary fibrosis [21]. The heterogeneity and origin of lung cell lines during lung injury has been an important research focus in recent decades [22]. In the current study, we utilized multiplex scRNA-seq technology to evaluate the expression profiles of lung cell compositions across normal, pathological, and high-pressure exosome intervention groups. Our research provides an in-depth understanding of the pathogenesis of alveolar damage by characterizing several pathological cell populations with distinct molecular expression profiles. Specifically, the scRNA-seq results revealed dynamic changes in lung cell compositions following exosome treatment, characterized by a significant reduction in inflammatory lymphocytes, including NK cells, CD8^+^ T cells, and CD4^+^ T cells, alongside a restoration of the alveolar epithelial cell populations (AT1 and AT2 cells). Furthermore, comprehensive gene expression analyses indicated a significant upregulation of inflammation pathways associated with oxidative stress, suggesting that these pathways may play a crucial role in the processes of lung injury and repair. These findings not only provide a new paradigm for understanding the pathological mechanisms of POT but also offer fresh insights for potential therapeutic interventions in the future.

The formation and repair of the alveolar gas exchange region requires the precise coordination of interactions among different epithelial, stromal, and immune cells [23]. In this study, we obtained a cellular picture of the recovery from lung injury after exosome treatment. Our focus was on abnormal epithelial cells, lymphoid lineage cells, and myeloid cells. Airway epithelial cells form an important innate barrier that effectively protects the lungs [24]. In response to chronic infection and inflammation, the epithelial layer is remodeled [25]. Airway cells may contribute to the pathogenesis of major chronic lung diseases, including chronic obstructive pulmonary disease, cystic fibrosis, asthma, and bronchial cancer. The alveolar epithelium is mainly composed of AT1 and AT2 cells. AT1 cells are responsible for effective gas exchange. AT2 cells, as alveolar stem cells, can self-renew and differentiate into AT1 cells [26]. Hyperoxia exposure was found to alter all cellular compartments, particularly the alveolar epithelium, stromal fibroblasts, the capillary endothelium, and macrophage populations. Among our data, analysis of the BPD-associated genes pointed to marked changes in expression in many of the cell populations sensitive to hyperoxia, underscoring the important roles played by these cell populations in the disease process. Pathway analysis and predicted dynamic cellular crosstalk suggested inflammatory signaling as the main driver of hyperoxia-induced change.

Human mesenchymal stem cell-derived exosomes (hMSC-Exos) are nanoscale extracellular vesicles (typically 30–150 nm in diameter) secreted by mesenchymal stem cells (MSCs) under both physiological and pathological conditions [27]. These exosomes are enriched with a variety of bioactive molecules, including proteins, lipids, mRNAs, and non-coding RNAs such as microRNAs (miRNAs) and long non-coding RNAs (lncRNAs), which are encapsulated within a lipid bilayer membrane [28,29]. hMSC-Exos serve as essential mediators of intercellular communication and play pivotal roles in modulating immune responses, promoting tissue repair, and maintaining cellular homeostasis [30]. They have been shown to exert therapeutic effects in various disease models, including inflammation, fibrosis, neurodegeneration, cardiovascular injury, and cancer, primarily through the regulation of target-cell gene expression, signal transduction pathways, and phenotypic modulation [31,32]. Due to their low immunogenicity, inherent biocompatibility, and ability to cross biological barriers, hMSC-derived exosomes have emerged as a promising cell-free therapeutic strategy. Compared with whole-cell transplantation, exosome-based therapies offer advantages in terms of safety, storage, scalability, and regulatory feasibility, thereby holding significant translational potential for clinical applications [33,34].

Exosome-like nanovesicles ameliorate lung inflammation associated with dextran sulfate sodium-induced colitis through modulating macrophage polarization [9]. Importantly, exposure to hyperoxia impairs the composition and expression patterns of all cellular compartments necessary for normal alveolarization [35]. The changes were gradual. Multiple affected transcriptional programs were related to the activation of the inflammatory response, suggesting that inflammation is one of the main drivers of hyperoxia-induced changes. Previously, fibroblasts (Col13^+^ and Col14^+^), SMCs, macrophages, AT1 cells, and EC lymph cells were predicted to exhibit the strongest network of interactions in lung homeostasis. Our analysis identified hyperoxia-induced interactions between cell compartments in the lung, further revealing the lung cell types most actively involved in the cellular crosstalk in hyperoxia and specifying the activated receptor pathways. We included selected analyses of interactions induced by hyperoxia. In addition, our dataset allowed for further systems-level analyses of homeostasis during normal development, though such analyses were not carried out in the present work. Notably, the efficacy of MSC-Exos reported here is in line with the results of clinical exosome trials in cases of pulmonary fibrosis [25]; however, our focus on hyperoxia-specific niches further advances the field. The limitations of the work reported here include the unexamined long-term effects and in vivo exosome biodistribution. Future studies should explore the miRNA cargo of MSC-Exos and investigate their translational potential.

## 4. Materials and Methods

### 4.1. Animals

Female C57BL/6 mice, each weighing about 18 g (Shanghai Bikai Laboratory Animal Company, Shanghai, China), were kept in the Navy Medical University Animal Center. Eighteen mice were randomly divided into three groups based on body weight, with six mice in each group: control group—This group served as a negative control, receiving no treatment, and was maintained under standard atmospheric conditions. To eliminate potential solvent effects, the mice were administered 100 μL of phosphate-buffered saline (PBS) via intravenous injection. Hyperbaric oxygen exposure group (POT group)—To ensure that the sole experimental variable was hyperbaric oxygen exposure, animals were intravenously injected with the same volume of PBS as that used in the control group, followed by exposure to a hyperbaric oxygen environment at a pressure of 0.20 MPa and an oxygen concentration of ≥95% for 8 h. Hyperbaric oxygen exosome intervention group (MSC-Exos + POT group)—Pharmacokinetic studies of exosomes indicate that peak concentrations in lung tissue occur 24 h following intravenous injection [36]. To ensure effective action of exosomes during treatment, and in accordance with prior research, we administered 100 μg of exosomes [37] (at a concentration of 1 μg/μL) via intravenous injection one day prior to hyperbaric oxygen exposure, followed by the same hyperbaric oxygen treatment as in the POT group.

### 4.2. Isolation and Characterization of Human Umbilical Cord Mesenchymal Stem Cell-Derived Exosomes (hUMSC-Exos)

Serum-free conditioned medium from human umbilical cord mesenchymal stem cells (hUMSCs) was collected. Cells and debris were removed using sequential centrifugation at 300× *g*, 2000× *g*, and 10,000× *g*. The processed supernatant was used as the feed material for tangential flow filtration (TFF) with a 300 kDa molecular weight cutoff (MWCO) membrane cassette. The concentrated solution was collected and further centrifuged at 100,000× *g* for 70 min. The resulting pellet was then resuspended in PBS, aliquoted, and stored at −80 °C for characterization and subsequent experiments. The morphology was observed using transmission electron microscopy (TEM). The nanoparticle size distribution was analyzed using nanoparticle tracking analysis (NTA). Western blot analysis was performed to confirm the expression of the exosomal markers CD63 and CD9. The exosomal protein concentration was quantified using a BCA assay kit.

### 4.3. Characterization of Exosomes

Exosomes were analyzed for morphology using cryo-transmission electron microscopy (Cryo-TEM). First, grids (Quantifoil, R2/2, 200 mesh, MiTeGen, New York, NY, USA) were hydrophilically treated using a glow discharge system (PELCO easiGlow, Ted™ Pella, Redding, CA, USA). Subsequently, 4 μL of the sample was added to the treated grid and dried for 1.5 s under conditions of 4 °C and 100% humidity. The sample was then rapidly frozen in liquid ethane, with vitrification performed using a Vitrobot Mark IV (Thermo Fisher Scientific, Waltham, MA, USA). Finally, the sample was analyzed using a Talos L120C transmission electron microscope (Thermo Fisher Scientific, Waltham, MA, USA) at 120 kV. Additionally, nanoparticle analysis was conducted using a NanoSight 300 (Malvern Instruments, Malvern, UK) to determine the size and concentration of the exosomes.

### 4.4. Histological Analysis

Formalin-fixed, paraffin-embedded lung sections of 2 μm thickness were stained with H&E (Masson and Sirius Red), and their histological scores were determined. A lung pathologist assessed the severity of alveolar septal fibrosis. Scoring was performed in a blinded fashion in ten consecutive fields at a magnification of 400× per section. All tests were repeated three times. H&E staining was used to assess inflammation and fibrosis.

### 4.5. Quantitative Real-Time Polymerase Chain Reaction (Qrt-PCR) Analysis

Lung tissues were weighed and homogenized with TRIzol lysis reagent (volume adjusted according to tissue weight). The homogenate was centrifuged at 1000× *g* for 10 min at 4 °C, and the supernatant was collected. Total RNA was extracted using the phenol-chloroform method, and the RNA concentration was quantified. The mRNA levels of interleukin-6 (*IL-6*), and C-X-C motif chemokine ligand 10 (*CXCL10*) in lung tissues were detected using PCR. β-Actin (*ACTB*) was used as an internal reference, and relative expression levels were calculated using the 2^−ΔΔCT^ method. Initial denaturation at 95 °C for 5 min was followed by 45 cycles of 5 s at 95 °C (denaturation); 10 s at 58 °C (annealing); and 20 s at 72 °C (extension).

### 4.6. Western Blot Analysis

Lung tissue lysates were mixed with 5× loading buffer and boiled at 100 °C for 5 min. A 20 μL aliquot of each sample was loaded onto an SDS-PAGE gel for electrophoresis. Proteins were transferred to a nitrocellulose membrane at 300 mA constant current. The membrane was blocked with 5% skim milk at room temperature for 1 h. According to the guidelines issued by the International Society for Extracellular Vesicles (ISEV) (MISEV2018), the characterization of exosomes requires at least three transmembrane or cytosolic markers (such as CD9, CD81, TSG101) and one negative marker (such as Calnexin). The membrane was incubated overnight at 4 °C with primary antibodies against CD81, CD9, TSG101, and calnexin. After three washes with TBST (15 min each), the membrane was incubated with a horseradish peroxidase (HRP)-conjugated secondary antibody at room temperature for 1 h, followed by three additional TBST washes (15 min each). Signals were developed using enhanced chemiluminescence (ECL) reagent and captured using an imaging system.

### 4.7. Enzyme-Linked Immunosorbent Assay (ELISA)

Fresh mouse lung tissue was homogenized in RIPA lysis buffer containing PMSF and proteinase inhibitors and then centrifuged at 1000× *g* for 30 min at 4 °C. The total protein concentration was measured using the BCA assay kit (Catalog No. P0012S, Beyotime, Shanghai, China), and samples were normalized accordingly. Standard solutions were prepared as per the Interleukin-1 beta (IL-1β) (Catalog No. PI301, Beyotime, Shanghai, China) and Tumor necrosis factor-alpha (TNF-α) (Catalog No. PT512, Beyotime, Shanghai, China) assay kit instructions. Samples were added to antibody-coated microplate wells, followed by biotin-labeled antigens, and incubated at 37 °C for 60 min. After washing with PBST five times, horseradish peroxidase-conjugated streptavidin was added. The plate was then incubated again at 37 °C for 20 min, followed by another five washes. TMB substrate was added for color development, the incubation was then carried out in the dark for 20 min, and finally, it was stopped using sulfuric acid. The absorbance was measured at 450 nm using a microplate reader, and the sample concentrations were calculated using a logistic curve (four-parameter fit) derived from the standard curve.

### 4.8. Preparation of Single-Cell Suspensions

The lung tissue was weighed and added to the corresponding body weight. TRIzol lysate was prepared into a homogenate, and tissue samples were chopped into cubes of about 1 mm^3^, digested, and prepared as a single-cell suspension using the Multi Tissue dissociation kit (Miltenyi Biotec, Cat# 130-110-201, Bergisch Gladbach, Germany). The 0.25 g tissue sample was digested with 50 μL of enzyme D, 35 μL of enzyme R, and 10 μL of enzyme A in 1 mL of RPMI and then incubated at 37 °C for 30 min. The reaction was deactivated by adding 10% FBS. The solution was then passed through a 40 μm cell strainer. After centrifugation at 1000 RPM for 5 min, the cell pellet was incubated with 1 mL of RBC lysis buffer on ice for 3 min. After washing the single cells with PBS, the cell count was analyzed using the Countess automated cell counter, and cell viability was assessed using the Trypan Blue exclusion method.

### 4.9. Data Quality Control and Preprocessing

In this study, we utilized microfluidic chip technology to isolate and capture single cells, encapsulating cells or cell nuclei within a micro-reaction system formed by oil droplets (Gel Beads in Emulsion, GEMs). Each GEM contains either a single cell or cell nucleus along with gel beads carrying barcodes and primers. Following cell lysis, the released mRNA is reverse transcribed to generate barcoded cDNA, achieving single-cell labeling. Subsequently, the GEMs are disrupted, and single-stranded cDNA is purified and enriched using magnetic beads, followed by PCR amplification to obtain sufficient amounts of cDNA. The quality-controlled amplified products then enter the library construction process, which includes fragmentation, adapter ligation, and sample index PCR, ultimately producing a library that meets sequencing requirements. The library is sequenced using the Illumina NovaSeq 6000 platform for high-throughput sequencing in PE150 mode, with a sequencing depth of approximately 50,000 reads per cell to ensure data coverage and accuracy. The generated raw data undergo preliminary analysis using the official 10× Genomics’ Cell Ranger software package (version 3.1.0). This includes aligning sequencing reads to the reference genome, generating a gene expression matrix, and filtering out low-quality cells and doublet droplet data. After constructing the gene–cell data matrix, we excluded cells of poor quality, such as those expressing fewer than 200 unique genes or more than 3000 genes, as well as cells with mitochondrial gene percentages exceeding 50%. The data are transformed using the natural logarithm and normalized to standardize the sequencing depth to 10,000 molecules per cell. We then regress out the number of unique molecular identifiers (UMIs) using the Seurat package and apply batch effect correction with edgeR to ensure the accuracy of the analysis.

### 4.10. Dimensionality Reduction and Clustering Analysis Based on Single-Cell Sequencing Data

The Seurat R package (version 1.4.0.5) was used for dimensionality reduction analysis. We first identified highly variable genes across the single cells, after controlling for the relationship between the average expression and dispersion. Genes were placed into 20 bins based on their average expression and removed using a 0.0125 low cutoff and a 0.3 high cutoff [38]. Within each bin, a z-score of log-transformed dispersion measure (variance/mean) was calculated. A z-score cutoff of 0.5 was applied to identify the highly variable genes [39], resulting in a total of 1140 genes. We then performed Principal Component Analysis (PCA) using the variable genes as input and determined the significant Principal Components (PCs) using the jackStraw function in the Seurat package. A total of 20 statistically significant PCs were selected as input for t-distributed stochastic neighbor embedding (tSNE). tSNE visualized the single cells in a two-dimensional space based on the expression signatures of the variable genes, reflecting cell subpopulation heterogeneity consistent with the PCA-driven clustering patterns.

### 4.11. Identification of Differentially Expressed Genes and Marker Genes

Cell-specific marker genes were identified in two stages. The first sets of differentially expressed genes (DEGs) were identified by comparing cells in a specific cluster with cells in all other clusters (Seurat package likelihood-ratio test: average expression difference > 0.5 natural log with an FDR-corrected *p* < 0.01). Next, cells in a specific cluster were compared with cells in every other cluster in a pairwise manner to identify a second set of DEGs (Seurat package likelihood-ratio test: average expression difference > 0.25 natural log with *p* < 0.05). Cell-specific markers were identified by overlapping the first and second sets of DEGs. Because different cells in the lung share some well-known markers (transitional cells vs. intercalated cells, and proximal tubule cells vs. novel cells), the combination of these two approaches using the lower threshold enabled us to retain the shared markers while identifying markers distinct from those of other cells.

### 4.12. Cell Clustering Analysis

We utilized the SingleR (version 1.6.1) tool (https://github.com/dviraran/SingleR (accessed on 5 November 2024)) to annotate and assign cell types by comparing the gene expression profiles from our single-cell RNA sequencing (scRNA-seq) data with the ImmGen dataset [40]. A density-based spatial clustering algorithm (DBSCAN) was used to identify cell types on the tSNE map with an initial setting of an eps value of 0.5 [41]. Clusters were removed if their number of cells was less than 10. The remaining cells were clustered again with an eps value of 1, and any cluster whose number of cells was less than 20 was removed. After pruning, we removed 320 cells (1.1% of our data), and 27,424 cells were used for further analysis. In a post hoc test of the final 16 clusters, every pair was found to have more than 10 differentially expressed genes (average expression difference > 1 natural log, with FDR-corrected *p* < 0.01). We used the same procedure, with modifications, for subclustering. DBSCAN was then used to identify cell types on the tSNE map with an initial eps value of 0.5. Briefly, the following six steps were carried out: ① preparation of single lung cell suspension; ② single-cell RNA sequencing—library construction and quality control; ③ data quality control and preprocessing; ④ dimensionality reduction and tSNE visualization; ⑤ identification of differentially expressed genes and marker genes; and ⑥ cell clustering analysis—dimensionality reduction analysis and the tSNE map showed that lung cells were divided into 12 clusters.

### 4.13. Statistical Analysis

All experiments were performed at least three times for each group, and statistical analyses were performed using GraphPad Prism Software (version 7). The results are presented as mean values ± standard deviation. For statistical analysis, the following methods were applied: one- and two-way analysis of variance (ANOVA) for multiple groups, and Student’s *t*-test for two groups. Survival time was analyzed using the Kaplan–Meier method and log-rank testing. A *p*-value < 0.05 indicated statistical significance.

## 5. Conclusions

This study enhances our understanding of the pathogenic mechanisms underlying hyperbaric oxygen-induced lung injury and highlights the therapeutic potential of stem cell-derived exosomes in targeting specific molecular and cellular adaptations. Importantly, it provides a promising avenue for developing tailored interventions that address the unique challenges posed by the pathological microenvironment of hyperbaric oxygen toxicity. The insights into the role of these exosomes in modulating disease progression—particularly in responses to metabolic stress and inflammation—may inform the development of novel strategies to slow or even reverse the advancement of hyperbaric oxygen injury in patients. Nevertheless, the findings presented here require additional functional validation and further clinical studies to confirm their efficacy and applicability in therapeutic settings.

## Data Availability

All data are present in the manuscript. The data that support the findings of this study are available from the corresponding author upon reasonable request.
